# Diet Significantly Influences the Immunopathology and Severity of Kidney Injury in Male C57Bl/6J Mice in a Model Dependent Manner

**DOI:** 10.3390/nu13051521

**Published:** 2021-04-30

**Authors:** John E. Brus, Daniel L. Quan, Kristin J. Wiley, Brittney Browning, Hannah Ter Haar, Riley Lutz, Jeffrey F. Houghton, Joseph C. Gigliotti

**Affiliations:** Department of Integrative Physiology and Pharmacology, Liberty University College of Osteopathic Medicine, Lynchburg, VA 24502, USA; jebrus@liberty.edu (J.E.B.); dlquan@liberty.edu (D.L.Q.); kjwiley1@liberty.edu (K.J.W.); brittneydawn9@gmail.com (B.B.); hjt6507@uncw.edu (H.T.H.); rlutz1@liberty.edu (R.L.); jhoughton6@liberty.edu (J.F.H.)

**Keywords:** western diet, mouse, acute kidney injury, dietary quality, nutrition

## Abstract

Diet is a leading causative risk factor for morbidity and mortality worldwide, yet it is rarely considered in the design of preclinical animal studies. Several of the nutritional inadequacies reported in Americans have been shown to be detrimental to kidney health; however, the mechanisms responsible are unclear and have been largely attributed to the development of diabetes or hypertension. Here, we set out to determine whether diet influences the susceptibility to kidney injury in male C57Bl/6 mice. Mice were fed a standard chow diet, a commercially available “Western” diet (WD), or a novel Americanized diet (AD) for 12 weeks prior to the induction of kidney injury using the folic acid nephropathy (FAN) or unilateral renal ischemia reperfusion injury (uIRI) models. In FAN, the mice that were fed the WD and AD had worse histological evidence of tissue injury and greater renal expression of genes associated with nephrotoxicity as compared to mice fed chow. Mice fed the AD developed more severe renal hypertrophy following FAN, and gene expression data suggest the mechanism for FAN differed among the diets. Meanwhile, mice fed the WD had the greatest circulating interleukin-6 concentrations. In uIRI, no difference was observed in renal tissue injury between the diets; however, mice fed the WD and AD displayed evidence of suppressed inflammatory response. Taken together, our data support the hypothesis that diet directly impacts the severity and pathophysiology of kidney disease and is a critical experimental variable that needs to be considered in mechanistic preclinical animal studies.

## 1. Introduction

Lifestyle is a leading contributor to overall wellness and significantly influences the outcomes of cardiovascular disease (CVD) regardless of genetic risk [1]. Diet is a major component of lifestyle, and poor diet and diet-associated diseases account for more than eight of the top 30 risk factors for morbidity and mortality worldwide [2]. However, the mechanisms describing how poor nutrition negatively impacts health remain surprisingly unclear.

A major barrier in nutrition research has been the difficulty in appropriately modeling the complex and multifaceted nature of human dietary habits in mechanistic preclinical animal studies. Chow diets are the most-used food source in preclinical rodent studies due to their high nutritional quality, storage stability, and relatively low manufacturing costs. Composed of grain-based mixtures that are supplemented with high-quality oils and vitamin/mineral-mixtures, chow diets are poor representations of the relatively low nutritional quality reported in developed societies. This has led to the creation of a variety of disease-inducing diets, most of which focus on the high-caloric intake of developed nations and produce obesity and metabolic dysfunction in wild-type animals. Although obesity and metabolic dysfunction remain major health concerns worldwide, current commercially available high-calorie diets represent an extreme and very narrow view of the complex nutritional inadequacies reported in Western societies. The physiological effects of a given nutrient are often dependent upon the existence and abundance of other nutrients (such as the sodium-potassium ratio [3,4]) and these interactions are not considered in commercially available disease-inducing diets. Therefore, it is possible that the reductionist approach that is common in nutrition research has unintentionally hindered the successful translation of mechanistic data from rodents to humans.

In an attempt to address this issue, we are developing a novel Americanized diet (AD) that includes several of the nutritional inadequacies reported in Americans [5]. The AD is a two-pellet diet that consists of a grain-based pellet and a synthetic “sweet and salty” pellet and was developed with the interest of studying dietary preference and may offset reported adverse effects of traditional high-calorie rodent diets [6]. Interestingly, several of the nutrient modifications made in the AD have been suggested to impact kidney health in either a direct or indirect manner [7,8,9,10]. Kidney dysfunction is a leading cause of CVD and a common outcome in diet-induced diseases such as diabetes and hypertension. Recent analyses have shown that poor dietary quality increases the incidence of kidney injury and disease [11]; however, the mechanisms responsible remain unclear. Therefore, we set out to determine if diet significantly impacts the outcomes of two different mouse models of kidney injury.

## 2. Materials and Methods

### 2.1. Animals and Diets

All animal experiments were performed following protocols approved by the Liberty University Institutional Animal Care and Use Committee and in accordance with the Guide for the Care and Use of Laboratory Animals [12]. Weanling (3-week-old) male C57Bl/6 mice were purchased from the Jackson Laboratory and group-housed (3–5 mice per cage) with corn cobb bedding, a standard rodent chow (Teklad Global 18%, #2018), and carbon-filtered tap water. After a 1-week acclimation period, mice were given ad libitum access to one of three diets: chow (Teklad Global 18%, #2018, *n* = 14), a commercially available Western diet (“WD,” #TD.88137, *n* = 13), or our novel “AD” (*n* = 14).

The AD consists of two-pellets, referred to as ADchow (TD.170651) and ADsynthetic (TD.170698 & TD.170699). The ADchow pellet is a modified chow-based pellet with reduced fat soluble vitamins (A, D, and E) and insoluble fiber (neutral detergent fiber). The ADsynthetic diet was formulated to include excess sucrose, fructose, saturated fats, cholesterol, omega-6 polyunsaturated fatty acids, *trans*-fatty acids, sodium, and phosphorus while having reduced potassium and omega-3 polyunsaturated fatty acids. Trans-fatty acids were included in the formulation as they are still consumed from natural sources (such as beef), but the inclusion in the ADsynthetic pellet was far lower than that used in other previous studies [13,14,15,16]. The inclusion for each nutrient was based on matching the percent difference of actual and recommended intake in mice and humans (depicted in Appendix A Table A1) based on recent reports by the United Stated Department of Agriculture [5,12]. The nutrient composition for each pellet was based on the calculated percent difference of reported and recommended intake values of each nutrient in Americans, and this percent difference was then applied to mouse nutrient requirements [13]. The final formulation of each pellet was then further adjusted to account for an average daily consumption of 2 and 0.5 g of ADsynthetic and ADchow, respectively. Another synthetic pellet, ADsythetic2, was formulated to accommodate for a change in diet preference beyond 8 weeks, where mice were consuming approximately equal amounts (1.5 g) of both ADchow and ADsynthetic2 daily. A summary of the diets used in this study is provided in Table 1.

### 2.2. Mouse Models of Acute Kidney Injury (AKI)

Mice remained on their experimental diets for 12 weeks prior to the induction of kidney injury with folic acid-induced nephropathy (FAN) or unilateral ischemia-reperfusion injury (uIRI) models. These models were chosen as they are routinely used in the literature and induce robust kidney injury in a dose-dependent manner [17]. The FAN model was performed based on previous publications [18,19,20,21] with modifications. Due to the differences in body weight between the dietary groups, we calculated the 250 mg/kg dose for the smallest mouse in our study (25 g) and administered the same dose (6.25 mg/500 µL) to all mice under brief isoflurane anesthesia (3% in 100% oxygen, 600 mL/min). Control mice (*n* = 4 for each diet) received an equal volume (500 µL) of 0.3 mM sodium bicarbonate vehicle as mice receiving the FAN (*n* = 4 for each diet). For the uIRI model, mice (*n* = 5–6 for each diet) were anesthetized with isoflurane and a flank incision was made and the artery and vein of the left kidney were isolated from surrounding connective and adipose tissue and obstructed with a microvascular clamp. After 45 min, the clamp was removed and restoration of blood flow was visually confirmed. The musculature was closed with 4.0 silk suture and the skin incision was closed with surgical staples. Surface body temperature was maintained at 28–30 °C with heat lamp and heating pad (normal surface body temperature ~30 °C). Buprenorphine (0.1 mg/mL, 100 µL subcutaneous injection) was administered as an analgesic once the animal began to regain consciousness.

### 2.3. Animal Euthanasia, Histological Analysis, and Serum Measures of Kidney Function

Animals were euthanized 24 (uIRI) or 48 h (FAN) later by cervical dislocation while under brief isoflurane anesthesia. The heart was removed and trunk blood was collected, left at room temperature for 10 min, and underwent centrifugation for 10 min at 2500 g. Serum was collected and frozen at −80 °C until analyzed for creatinine (Crystal Chem), blood urea nitrogen (BUN, Arbor Assays), or interleukin-6 (IL6, Biolegend) using commercially available assays. The kidneys were dissected and decapsulated, and a center sagittal section was fixed in 10% phosphate buffered formalin, embedded in paraffin, and 5 µm sections were cut and stained with H&E or Picro Sirius Red (ABCAM). In H&E-stained sections, the severity of acute tubular necrosis was assessed by an individual blinded to experimental design based on a 0–4 scale, where (0) refers to no evidence of injury, (1) is <25% of the identifiable tissue area consists of injury, (2) 25–50%, (3) 50–75%, and (4) >75%. Similar to previous work [22], “tubular injury” consisted of tubular dilation, proteinaceous casts, and apparent tubulointerstitial remodeling. The degree of tubulointerstitial fibrosis was assessed in Picro Sirius stained sections.

### 2.4. Nephrotoxicity PCR Array

Approximately 30–100 mg pieces of decapsulated kidney tissue were stored in RNAlater™ solution (Thermo). A single representative sample from each diet group in the FAN study was chosen that closely resembled the group average for serum creatinine, BUN, and histology scores in each treatment group. The selected sample was then processed to quantify the expression of 84 genes related to nephrotoxicity using the RT^2^ Profiler^TM^ PCR Array—Mouse Nephrotoxicity (#PAMM-094Z) as recommended by the manufacturer (Qiagen). Total RNA was isolated using the RNeasy^®^ kit, genomic DNA removed by DNA digestion, and 0.5 ug of total RNA was used for cDNA synthesis using the RT^2^ First Strand Kit. The cDNA mix was mixed with the RT^2^ SYBR^®^ Green Mastermix and nuclease-free water and then applied to the RT^2^ plate and analyzed as recommended using a Lightcycler™ 96 instrument (Roche). The ΔCt values for each treatment group were calculated based on the expression of housekeeping genes using the following equation: 2^ (ΔC_T_), where ΔC_T_ = (housekeeping (GAPDH))-C_T_ (gene of interest)]. The percent difference in expression as compared to control mice (chow-fed mice with vehicle treatment) were calculated for each gene. To identify genes that are potentially influenced by FAN and diet, we modified the analysis provided by the manufacturer (Qiagen). Genes with a greater than 2-fold difference from control mice were identified and from this list we identified genes with a ≥25% difference between mice fed the WD and AD. Six genes were identified using this methodology and their kidney mRNA expression was quantified using conventional real-time RT-PCR.

### 2.5. RT-PCR for Genes Associated with Nephrotoxicity, Inflammation, and Tissue Remodeling

Kidneys and spleens were processed for RT-PCR using commercially available kits (Qiagen and BioRad) following the manufacturers recommendations. Intron-spanning primers for genes implicated in nephrotoxicity or leukocyte specific markers were purchased from BioRad or created using Primer3 software [23,24,25]. Primer information is provided in Appendix C Table A2. Then, 10 µL RT-PCR reactions were performed using iTaq Universal SYBR Green Supermix (BioRad) and ΔC_T_ values calculated for each gene. All data are presented as normalized to the average of the control groups for each kidney injury model (chow-fed mice with vehicle injected for FAN or contralateral kidney of chow-fed mice in uIRI).

### 2.6. Statistical Analysis

All studies were performed using a completely randomized design. In the FAN study, “Diet” and “Treatment” were the independent variables with “Diet” having the 3 levels (chow, WD, and AD), and “Treatment” having 2 levels (vehicle or FAN). Similar analyses were used in the uIRI study, where “Treatment” was determined by comparing the gene expression data in the ischemic kidney to the non-ischemic, contralateral control kidney. All data were reviewed prior to GLM analysis and outliers identified using the Explore function and analyzed using general linear model procedures in SPSS (IBM). Values were considered significant with *p* ≤ 0.05 with Tukey Post Hoc analysis, and a tendency was defined as *p* ≤ 0.1.

## 3. Results

### 3.1. Poor Diet Exacerbates Tissue Injury in FAN

Mice fed the WD had the greatest total body weight (37.4 ± 4.1 g) as compared to mice fed the AD (30.3 ± 1.0, *p* = 0.001) and mice fed chow (26.8 ± 1.1, *p* < 0.001). Adiposity also differed amongst the groups, where mice fed the WD had the highest epididymal fat pad weight (2.0 ± 0.6 g, *p* < 0.001) as compared to mice fed chow (0.4 ± 0.08, *p* < 0.001) and the AD (0.7 ± 0.3, *p* < 0.001). The increase in body and adipose weight observed in mice fed the AD was also greater that in mice fed chow (*p* = 0.001 and *p* < 0.001, respectively). The adipose tissue weights did not differ between mice in the vehicle or FAN treated groups (*p* = 0.6).

**Figure 1:**
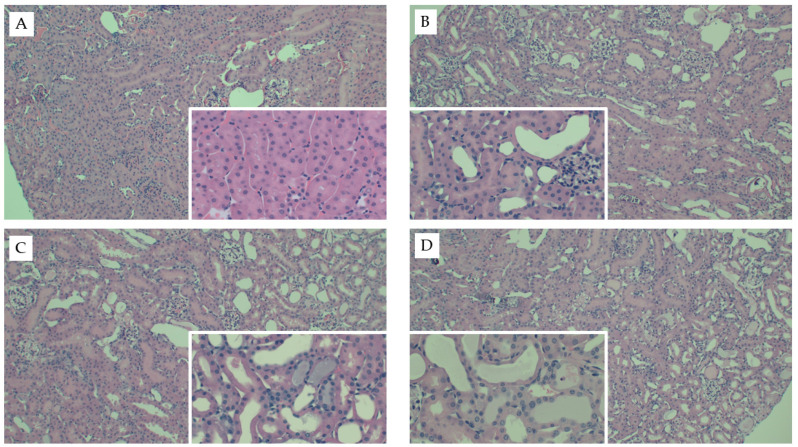
Representative kidney histology images from the FAN study. Representative H&E-stained kidney tissue images from mice fed chow and (**A**) 0.3 mM bicarbonate vehicle control, (**B**) folic acid injection (6.25 mg/500 µL, IP), (**C**) mice fed WD and administered folic acid (6.25 mg/500 µL, IP), or (**D**) mice fed AD and administered folic acid (6.25 mg/500 µL, IP). Larger images were captured at 10× magnification and inset images were captured at 40×.

**Figure A1:**
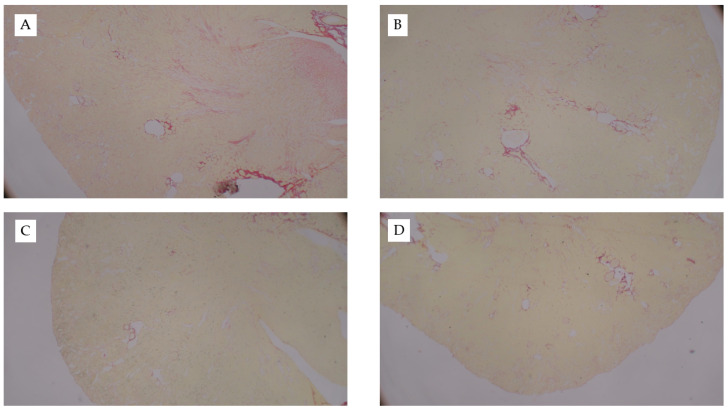
Picro Sirius-Stained Kidney Sections from FAN Study. Representative images of Picro Sirius-stained kidney tissue from mice fed chow and (**A**) 0.3mM bicarbonate vehicle control, (**B**) folic acid injection (6.25 mg/500 µL, IP), (**C**) mice fed WD and administered folic acid (6.25 mg/500 µL, IP), or (**D**) mice fed AD and administered folic acid (6.25 mg/500 µL, IP). Images captured at 10×.

Interestingly, mice fed the AD had the greatest kidney weights as compared to both mice fed chow (*p* < 0.001) and WD (*p* = 0.01) (Table 2). Kidney weights did not differ between mice fed chow or WD (*p* = 0.1) and these data were consistent when body weight was used as a covariate (*p* = 0.52). Circulating creatinine (*p* < 0.001) and BUN (*p* < 0.001) were significantly elevated in response to FAN, yet neither were significantly influenced by diet (*p* = 0.74 and 0.67, respectively). Diet did, however, significantly influence the severity of tissue injury assessed by microscopy with mice fed the WD (*p* = 0.001) and AD (*p* = 0.004) having more severe tissue injury as compared to mice fed chow (Table 2 and Figure 1). The predominate histological features of kidney injury were tubular dilation and acute tubular necrosis, which were more severe and widespread throughout the cortex in mice fed the AD and WD. No difference in renal fibrosis was observed between the diet groups in Picro Sirius stained sections (Appendix C Figure A1). FAN caused a significant (*p* = 0.005) increase in circulating interleukin-6 (IL6) that was further exacerbated with WD as compared to mice fed the AD (*p* = 0.01) and chow (*p* = 0.006, Table 2). Likewise, FAN caused a drastic increase in renal IL6 mRNA expression (*p* < 0.001); however, there was no significant difference among the diet groups (*p* = 0.9).

### 3.2. Diet Significantly Influences the Renal mRNA Expression of Nephrotoxic and Leukocyte-Specific Genes in FAN

**Figure 2:**
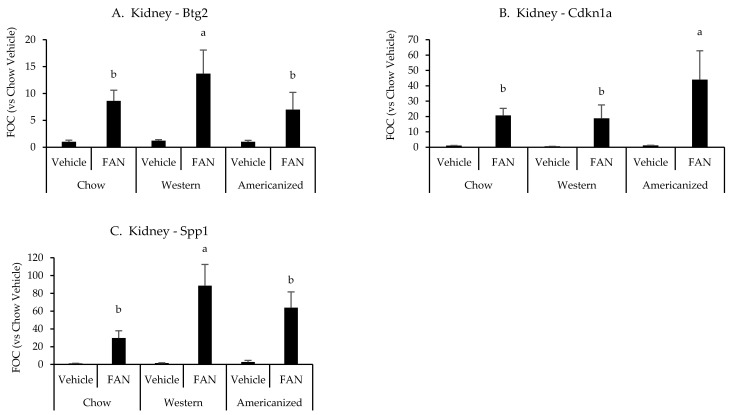
Kidney gene expression data from the diet-FAN study. Data are presented normalized to chow-fed mice with vehicle treatment (average + STD). (**A**) Kidney—*Btg2*, (**B**) Kidney—*Cdknla*, (**C**) Kidney—*Spp1.* Different superscript letters (a vs. b) signify significant difference between diets (*p* < 0.05) as determined by Two-Way ANOVA with Tukey Post Hoc test. Treatments with similar superscript letters are not statistically different (*p* > 0.05). RT-PCR data and statistics are presented in Appendix F Table A4. *n* = 4 for each diet-treatment combination. FOC—fold change.

We next set out to identify potential mechanisms responsible for the exacerbation of tissue injury in mice fed the WD and AD. Representative samples from each diet were selected and the expression of 84 genes associated with nephrotoxicity were screened using a commercially available PCR array (raw data are presented in Appendix E Table A3). As compared to vehicle-treated chow-fed mouse, 57 nephrotoxic genes were identified as having a greater than 50% change in expression with FAN and six of these were identified as differing based on diet (Appendix F Table A4). Of the six genes identified by the nephrotoxicity assay, kidney expression of *Btg2* (*p* = 0.03)*, Cdkn1a* (*p* = 0.01) and *Spp1* (*p* = 0.001) were significantly influenced by diet as determined using conventional RT-PCR. Mice fed the WD had greater expression of *Btg2* and *Spp1* as compared to mice fed chow and the AD, while mice fed the AD had a greater expression of *Cdkn1a* as compared to mice fed chow and the WD (Appendix F Table A4 and Figure 2). We also quantified the renal mRNA expression of 4 leukocyte-specific markers (*CD3e*, *CD19*, *CCR2*, and *Ly6G*), all of which were significantly influenced by FAN (Appendix F Table A4). However, diet had no significant effect on the expression of leukocyte specific markers in the kidney.

**Figure 3:**
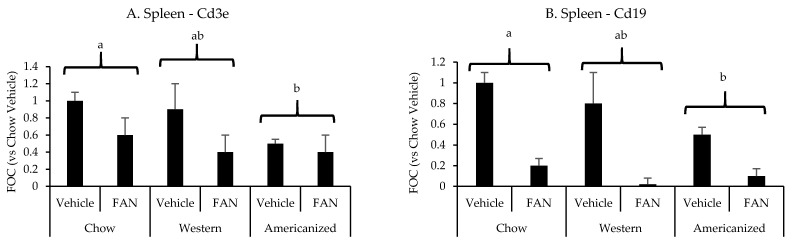
Spleen leukocyte-specific gene expression data from the diet-FAN study. Data are presented normalized to chow-fed mice with vehicle treatment (average + STD). (**A**) Spleen—*Cd3e*, (**B**) Spleen—*Cd19*. Different superscript letters (a vs. b) signify significant difference between diets (*p* < 0.05) as determined by Two-Way ANOVA with Tukey Post Hoc test. Treatments with similar superscript letters are not statistically different (*p* > 0.05). RT-PCR data and statistics are presented in Appendix F Table A4. *n* = 4 for each diet-treatment combination. FOC—fold change.

We have previously shown the spleen to significantly influence the immunopathology of kidney injury [26,27,28]. Therefore, we set out to determine if diet modifies the splenic mRNA expression of the same leukocyte-specific markers in FAN. FAN caused a significant decrease splenic mRNA expression of the T-cell marker *Cd3e* (*p* = 0.001) and the B-cell marker *Cd19* (*p* < 0.001) (Figure 3 and Appendix F Table A4). Regarding diet, mice fed the AD had lower splenic mRNA expression of *Cd3e* (*p* = 0.01) and *Cd19* (*p* = 0.009) as compared to mice fed chow but not mice fed the WD (*p* ≥ 0.2). There was no significant effect of diet (*p* = 0.8) or FAN (*p* = 0.1) on splenic *Il6* expression (data not shown).

### 3.3. Diet Significantly Influences Leukocyte-Specific Gene Expression in uIRI

**Figure 4:**
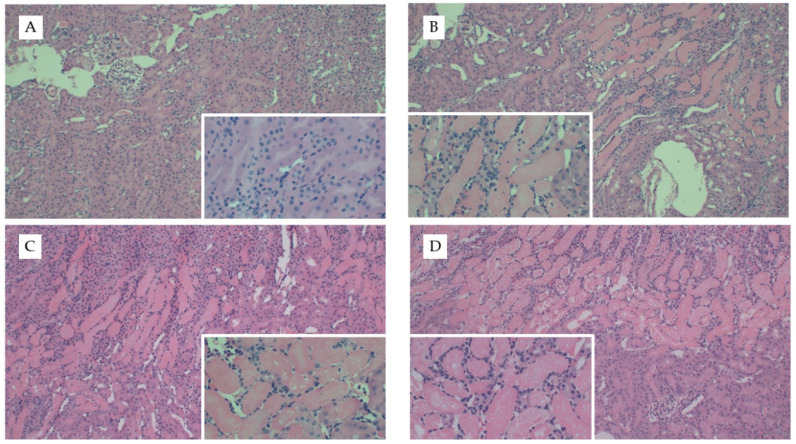
Representative kidney histology images from the uIRI study. Representative images from H&E stained kidney tissue from mice fed chow and (**A**) healthy control kidney, (**B**) uIRI, (**C**) mice fed WD and uIRI, or (**D**) mice fed AD and uIRI. Inset images show (**A**) normal renal tubular structure as compared to the (**B**–**D**) proteinaceous casts and acute tubular necrosis with uIRI. Larger images were captured with 10× and inset images were captured at 40×.

**Figure A2:**
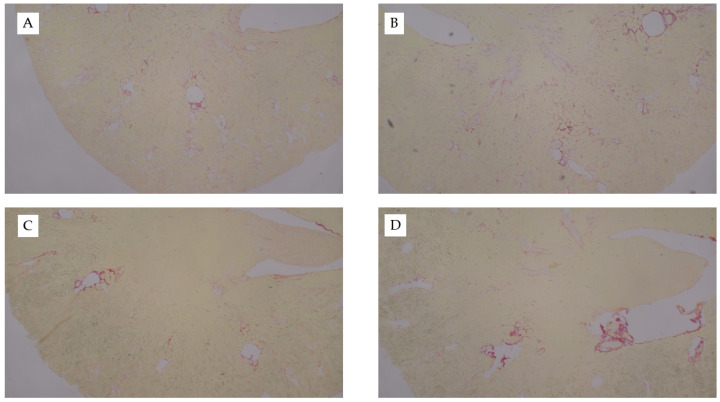
Picro Sirius-stained kidney sections from uIRI study. Representative images of Picro Sirius-stained kidney tissue from the diet-uIRI study. (**A**) Contralateral control kidney of chow-fed mouse, (**B**), ischemic kidney from chow-fed mouse, (**C**) ischemic kidney from WD-fed mouse, or (**D**) ischemic kidney from AD-fed mouse. Images captured at 10×.

We repeated our diet studies in the uIRI model to assess the generalizability of the FAN data. Despite the significant difference in adiposity between dietary treatment groups, there were no significant differences in surgical time (~5 min) between treatment groups (*p* = 0.5). Diet had no effect on the severity of tissue injury as determined by quantification of H&E- (*p* = 0.7, Figure 4) or Picro Sirius-stained slides (Appendix D Figure A2). Whereas AD-fed mice had greater kidney weights in the FAN study, there was only a tendency for diet to influence kidney weights (*p* = 0.07) in the uIRI study. In the nonischemic contralateral kidney, mice fed chow had lower numerical kidney weights (160 ± 22 mg) as compared to mice fed the AD (168 ± 14) and WD (186 ± 15). Similar numerical trends were observed in the ischemic kidneys where mice fed chow (173 ± 24 mg) or AD-fed mice (170 ± 12) had lower numerical kidney weights compared to mice fed the WD (195 ± 32). These data suggest that the effect of diet on kidney weight with injury is dependent upon the model utilized.

**Figure 5:**
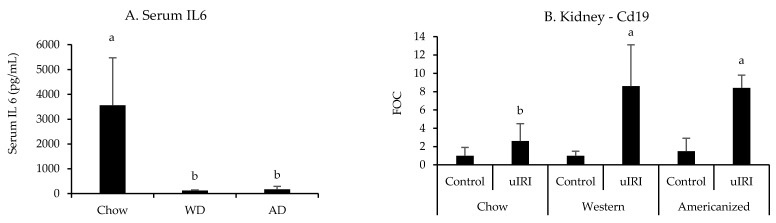
Circulating IL6 and renal *Cd19* mRNA expression following uIRI. RT-PCR data are presented normalized to the healthy contralateral kidney in chow-fed mice (average + STD). Different superscript letters (a vs. b) signify significant difference between diets (*p* < 0.05) as determined by Two-Way ANOVA with Tukey Post Hoc test. Treatments with similar superscript letters are not statistically different (*p* > 0.05). RT-PCR data and statistics are presented in Appendix G Table A5. *n* = 4–6 for each diet-treatment combination. FOC—fold change.

All nephrotoxic and leukocyte-specific genes quantified in the kidneys were significantly influenced by uIRI except for *Cd3e* (*p* = 0.1, Appendix G Table A5). However, none of the nephrotoxic genes identified in the FAN study were significantly influenced by diet. In regards to the expression of leukocyte-specific genes, the effect of uIRI on renal *Cd19* mRNA expression was highly dependent upon diet fed (*p* = 0.008), with mice fed the WD and AD having approximately four-fold higher renal *Cd19* expression after uIRI as compared to mice fed chow (Figure 5). There was also a tendency for mice fed chow to have a greater renal *Ly6g* expression after uIRI (*p* = 0.06, Appendix G Table A5). Interestingly, mice fed the WD (*p* = 0.006) and AD (*p* = 0.004) had significantly lower circulating IL6 concentrations as compared to mice fed chow (Figure 5).

**Figure 6:**
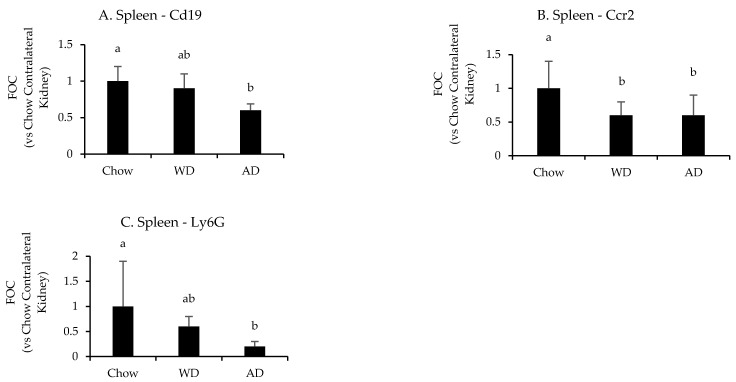
Splenic expression of leukocyte specific genes following uIRI. Data are presented normalized to chow-fed mice. Different superscript letters (a vs. b) signify significant difference between diets (*p* < 0.05) as determined by Two-Way ANOVA with Tukey Post Hoc test. Treatments with similar superscript letters are not statistically different (*p* > 0.05). RT-PCR data and statistics are presented in Appendix G Table A5. *n* = 4–6 for each diet-treatment combination. FOC—fold change.

Diet significantly affected the splenic mRNA expression of *Cd19* (*p* = 0.008), *Ccr2* (*p* = 0.004), and *Ly6g* (*p* = 0.03) following uIRI (Figure 6 and Appendix G Table A5). Mice fed chow had the highest splenic expression of these markers, with *Ccr2* than mice fed the WD (*p* = 0.02) and AD (*p* = 0.005), *Cd19* expression being significantly greater than mice fed the AD (*p* = 0.007), but not WD (*p* = 0.4), and greater *Ly6g* expression than mice fed the AD (*p* = 0.04) and a tendency for greater expression in mice fed the WD (*p* = 0.07). There were no differences in the expression of leukocyte specific markers between mice fed the WD or AD (*p* = 0.9, 0.8, and 0.1 for *Ccr2*, *Cd19*, or *Ly6g*, respectively).

## 4. Discussion

Here, we present data showing the effect of diet on the severity and immunopathology of kidney disease in preclinical mouse models. Mice fed the WD and AD both had elevated degrees of tissue injury and differential expression of nephrotoxic genes following FAN as compared to mice fed chow. Mice fed the WD, but not AD, also had significantly higher circulating levels of IL6 following FAN. In uIRI, we did not observe a significant effect of diet on the severity of tissue injury or the renal expression of similar nephrotoxic genes. However, we did observe a significant reduction in circulating IL-6 and the expression of leukocyte specific genes in the kidney and spleen of mice fed the WD and AD. Taken together, these data suggest that diet significantly influences the immunopathology of two separate mouse models of kidney injury.

There are surprisingly few studies addressing the effect of diet on outcomes of kidney injury. C57Bl/6 mice fed a high-fat diet for 9 weeks had worse cisplatin-induced kidney injury when compared to chow-fed mice [29]. It should be noted that the dose of cisplatin in this study was based on body weight and resulted in the high-fat mice receiving a higher absolute amount of cisplatin. This is a common complication in dietary studies and was the rationale for our administration of the same volume and absolute amount of FA to all mice.

Whereas there are a limited number of papers describing the effect of diet on outcomes of kidney disease, there is an appreciable literature available describing the effect of diabetes on the susceptibility and progression of kidney injury in rodents [30,31,32,33]. Ongoing (unpublished) studies in our lab suggest that mice fed the WD and AD have higher daily caloric intake (16 and 15 kcal/day, respectively) as compared to mice fed chow (12 kcal/day). Interestingly, only mice fed the WD display evidence of insulin resistance, with fasting hypoglycemia (120.3 mg/dL) observed as early as 8 weeks after diet initiation and developing a four-fold increase in fasting circulating insulin (4.2 ng/mL) after 24 weeks. Mice fed chow and the AD have almost identical fasting glucose (166 and 167 mg/dL, respectively) and insulin levels (0.6 and 1.0, respectively) at similar timepoints. Given the kinetics of these data, we believe the AD successfully models moderate nutritional inadequacies and is devoid of metabolic dysfunction in the timeframe of the current study, whereas mice fed the WD were likely developing insulin resistance.

Previous work has shown diabetic mice have altered renal hemodynamics [30], exacerbated inflammatory response [31,32], and altered expression of cell-cycle regulators [33] following kidney injury. Interestingly, we also observed alterations in the immune response and cell-cycle regulators in both WD- and AD-fed mice. Regarding the immune response, mice fed the WD had greater circulating IL6 concentrations than mice fed chow and the AD. This suggests that the overt obesity and developing metabolic dysfunction that is likely developing in the WD-fed mice exacerbates the immune response in FAN. We did not observe a significant increase in renal or splenic mRNA expression of IL6 in either the FAN or uIRI model suggesting another tissue source (perhaps liver [34]) is responsible. The spleen is a reservoir of deployable leukocytes [35,36] and we have previously shown the spleen to play a significant role in the pathophysiology of kidney injury with splenic size being inversely correlated with kidney injury [26,27,28]. Mice fed the WD and AD had >30% reduction in the expression of leukocyte markers in the spleen, which could be interpreted as there being an increased deployment of leukocytes from the spleen. This would suggest increased inflammation in the mice fed the WD and AD; however, the circulating IL6 data only supports this hypothesis in WD-fed mice. Additional studies with flow cytometry and broader characterization of circulating chemokines and cytokines are needed to better understand how diet influences the immunology and pathophysiology of FAN.

Regarding regulators of cell cycle progression, diet also significantly influenced the mRNA expression of *Btg2* and *Cdkn1a* (p21) following FAN. Knockout studies suggest a protective role for *Cdkn1a* in kidney disease (reviewed in [37]) and *Btg2* expression was also shown to be increased in the ischemic, but not contralateral control, kidney [38]. However, the function and significance of the upregulation of *Btg2* in kidney injury and disease has not been determined. *Btg2* is a downstream effector of NFκB and the cell cycle regulators p53 and p19 that result in cell cycle blockade using various cellular mechanisms (reviewed in [39]). Together, the current literature suggest that cell-cycle inhibitors are beneficial in reducing initial kidney injury, but may also enhance, or even facilitate, the subsequent progression to more chronic forms of disease [37,38,40,41,42,43]. The difference in expression of *Btg2* and *Cdkn1a* in the mice fed the WD and AD suggest morbid obesity and nutritional inadequacies influence the pathophysiology of kidney injury via different cellular mechanisms and may have different long-term outcomes. Furthermore, the differences in *Btg2* and *Cdkn1a* expression may contribute to the renal hypertrophy observed in the FAN model. The FAN model has been shown to induce an acute renal hypertrophy [20,21], and the AD has a dietary mineral composition that has also been shown to induce renal hypertrophy and injury in rats [44,45]. Therefore, it is possible that the altered mineral content (particularly the calcium and phosphorus) in the AD is responsible for the exacerbation of renal hypertrophy in FAN. Future studies are needed to verify this speculation and if *Btg2* and *Cdkn1a* are involved. 

We also found renal *Spp1* mRNA expression to be significantly influenced by both FAN and diet, with higher expression in mice fed the WD as compared to mice fed chow, but not AD. *Spp1* (osteopontin) is a glycoprotein that is normally secreted in the urine and is highly expressed in the kidneys, particularly in the distal cortical nephron. *Spp1* plays a key role in regulating calcium crystal formation and deposition within the kidney tubules [46,47,48,49], a process that is dependent upon diet, estrogen, and FGF23 [7,50,51]. It is generally upregulated following renal injury [52]; however, the role of *Spp1* in ischemic kidney injury is unclear and further studies are needed to elucidate the physiological significance of its increased expression with adiposity and disease.

We also assessed outcomes in a unilateral ischemia-reperfusion injury model. We did not observe changes in histology or the mRNA expression of the nephrotoxicity-associated genes identified in the FAN study in our uIRI model. The lack of difference in renal *Btg2* and *Cdkn1a* expression in the uIRI study may explain why kidney hypertrophy was not observed in mice fed the AD in the FAN study. However, there were effects of diet on the expression of immune markers in the kidney and spleen. The renal expression of the B-cell marker *CD19* was more than doubled in mice fed the WD and AD as compared to mice fed chow. The significance of this finding is unclear, as the role of B-cells in mouse models of ischemic AKI is complex and the outcomes are dependent upon several factors (subtype, antibody class, etc.) [53,54,55,56]. Diet also significantly influenced the mRNA expression of immune markers in the spleen (*Ccr2*, *Cd19*, and *Ly6g*), where mice fed the WD and AD had lower expression as compared to mice fed chow. As mentioned above, this could be interpreted as an increased deployment of leukocytes from the spleen and an exacerbation of the systemic immune response in mice fed the WD and AD. However, the suppressed circulating IL6 in mice fed the WD and AD would suggest an immunosuppressive effect of these diets in the uIRI model. Different molecular mechanisms of cellular injury in kidney disease models have been described [57,58,59], and our study suggests that diet may modify these mechanisms. Additional studies are needed to verify this speculation and determine how diet significantly modifies the inflammatory response and cell death pathways implicated in kidney injury.

There are limitations to consider when comparing our data to others in the literature, especially in the uIRI studies. We chose a unilateral model to reduce the complications we encountered while isolating the renal vasculature from the excessive adipose tissue in WD- and AD-fed mice. Therefore, serum measures of kidney function were not quantified in the uIRI studies given the presence of a healthy, non-ischemic contralateral kidney. We also used isoflurane anesthesia as obese mice did not tolerate the ketamine/xylazine anesthetic mixture that is commonly used in this model [60]. Isoflurane has been shown to reduce kidney injury in IRI [61] and required that we increase our ischemic time from 30 to 45 min to achieve the observed damage. These experiences caused concern about the outcomes of our uIRI studies and was the rationale for our focus on the FAN model. It is also important to highlight that changes in gene expression do not equate to changes in protein production, and additional studies are needed to validate changes in protein abundance and subsequent changes in cellular health and function.

## 5. Conclusions

In conclusion, we present data supporting the significance of diet on the susceptibility to kidney injury. Our findings suggest that a diet created to model the 50th percentile of nutrient intake in Americans is sufficient to exacerbate kidney injury in rodents to a similar degree as observed in a commercially available WD. Our data also suggest a mechanistic link between nutrition, inflammation, and cell-cycle regulation in the immunopathology of kidney injury that significantly alters disease outcomes. Taken together, our study highlights the need to increase the appreciation and consideration of nutrition in mechanistic preclinical animal studies.

## Figures and Tables

**Table 1:** Nutrient composition of experimental diets.

					Americanized Diet		
Parameter	Unit	AIN-93	Chow	Western	ADchow	ADsynthetic1	ADsynthetic2
Energy	kcal/gram	3.8	3.2	4.5	3.3	4.7	4.8
Protein	% Weight	17.7	18.6	17.3	16	19.4	19.4
Carbohydrates	% Weight	60.1	44.2	48.5	58.6	48.4	50.9
Sucrose + Fructose	Grams/kg	100	0	340	0	330	330
Cellulose/NDF	Grams/kg	50	151	50	75	20	0
Total Fat	% Weight	7.2	6.2	21.2	3.5	21.7	21.7
Saturated fatty acids	% Total Fatty Acids	15	0.6	64.5	15	41	41
ω6: ω3 PUFAs	Ratio	7:1	2:1	6:1	6:1	44:1	44:1
*trans*-fatty acids	% Total Energy	0	0	0	0	3	3
Cholesterol	Milligrams/kg	0	0	2076	0	600	600
**Minerals**							
Calcium	Grams/kg	5	10	6.8	9	5	5
Phosphorus	Grams/kg	3	4	5.4	3.4	9.6	8.7
Potassium	Grams/kg	3.6	6	3.6	6	2	0.4
Sodium	Grams/kg	1	2	1	2	4.6	4
**Vitamins**							
Vitamin A (RAE)	IU/kg	4000	15,080	26,292	9048	2250	0
Vitamin D (cholecalciferol)	IU/kg	1500	1500	2203	1500	375	0
Vitamin E (*RRR*-a-tocopherol)	IU/kg	171	111	121	171	42	0

NDF = neutral detergent fibers, PUFAs = polyunsaturated fatty acids, RAE = retinoic acid equivalents.

**Table 2:** Renal outcomes following FAN.

	Chow		WD		AD			
	Vehicle	FAN	Vehicle	FAN	Vehicle	FAN	*p* (FAN)	*p* (Diet)
Kidney Weights (mg)	292.5 ± 19.5	423.3 ± 22.5 ^b^	317.5 ± 19.5	495.0 ± 22.5 ^b^	412.5 ± 19.5	540.0 ± 19.5 ^a^	<0.001	<0.001
Serum Creatinine (mg/dL)	0.13 ± 0.1	2.4 ± 0.2	0.0 ± 0.1	2.7 ±0.2	0.0 ± 0.1	2.5 ± 0.1	<0.001	NS
BUN (mg/dL)	26.3 ± 10.6	227.7 ± 12.3	19.7 ± 10.6	215.0 ± 12.3	18.5 ± 10.6	227.9 ± 10.6	<0.001	NS
ATN Score (0–4)	0.5 ± 0.2	2.25 ± 0.2 ^b^	1.0 ± 0.2	3.3 ± 0.2 ^a^	1.0 ± 0.2	3.0 ± 0.2 ^a^	<0.001	0.005
Circulating IL-6 (pg/mL)	27.3 ± 13.3	784.3 ± 348.0 ^b^	24.0 ± 21.2	5043.0 ± 2692.4 ^a^	4.0 ± 5.3	724.5 ± 480.0 ^b^	0.005	0.004
Renal IL-6 mRNA (Relative)	1.0 ±0.8	683.3 ±274.2	1.8 ± 0.7	808.9 ± 563.5	3.8 ± 2.9	657.5 ± 657.7	<0.001	0.91

Values are presented as mean ± SEM, *n* = 4 for all groups. Different superscript letters (a vs. b) signify significant difference between diets (*p* < 0.05) as determined by Two-Way ANOVA with Tukey Post Hoc test. Treatments with similar superscript letters are not statistically different (*p* > 0.05). NS = no significant difference (*p* > 0.1).

## Appendix A

**Table A1:** Calculating target values for nutrients in experimental diets.

	Estimated Daily Intake ^$^			
Nutrient	Chow *	Western *	Americanized **	Human ***
Simple sugar (grams/kg)	0	240	92.5	120.0
Fiber (grams/kg)	202	0	−37	−47
Calcium (grams/kg)	100	36	18	−2.0
Phosphorus (grams/kg)	33	80	117	94
Potassium (grams/kg)	67	0	−23	−16
Sodium (grams/kg)	100	0	220	167
Vitamin A (RAE) (IU/kg)	277	557	−6	1
Vitamin D (cholecalciferol) (IU/kg)	0	47	−58	−51
Vitamin E (*RRR*-a-tocopherol) (IU/kg)	−35	−29	−59	−25

^$^ Estimated Daily Intake is presented as percent difference from estimated daily intake of the American Institute of Nutrition 1993 formulation (AIN93) based on citation [8]. * Estimates are based on mice consuming 3 g of chow or Western diet per day. ** Estimated based mice consuming 0.5 g of ADchow and 1.5 g of ADsynthetic1 per day for the first 6 weeks. After 8 weeks, estimates are based on mice consuming 1.5 g of each ADchow and ADsynthetic2. Final value is an average of calculated AD intake values using both ADsynthetic1 and ADsynthetic2. *** Human intake data are based on the “Usual Nutrient Intake from Food and Beverages” data derived from citation [5].

## Appendix B

**Table A2:** Primers Used for RT-PCR.

Gene (Abbreviation)	Sequence or BioRad Unique Assay ID
B-cell translocation gene 2 (*Btg2*)	qMmuCID0006843
Cyclin-dependent kinase 1a (*Cdkn1a*)	Forward—CAGACATTCAGAGCCACAG
	Reverse—GAGACAACGGCACACTTT
Secreted phosphoprotein 1 (Osteopontin) (*Spp1*)	qMmuCID0008550
TNF receptor superfamily member 12A (*Tnfrsf12a*)	Forward—CAATCATGGCTTCGGCTTGG
	Reverse—GGAGGTGCCTGGTGCTT
Clusterin (*Clu*)	qMmuCID0025019
Fibrinogen beta chain (*Fgb*)	qMmuCID0027043
C-C motif chemokine receptor 2 (*Ccr2*)	qMmuCID0016006
Cluster of Differentiation 3e (*Cd3e*)	qMmuCID0027036
Cluster of differentiation 19 (*Cd19*)	qMmuCID0017517
Lymphocyte antigen 6 complex, locus G (*Ly6g*)	qMmuCID0020071
Interleukin-6 (*Il6*)	qMmuCID0005613
Glyceraldehyde-3-phosphate dehydrogenase (*Gapdh*)	Forward—AACTTTGGCATTGTGGAAGG
	Reverse—GCAGGGATGATGTTCTGG

## Appendix E

**Table A3:** Raw RT^2^ Nephrotoxicity Array Data.

	Vehicle	FAN		
Gene	Chow	Chow	WD	AD
*A2m*	0.002176	0.00086	0.000647	0.000477
*Aass*	0.886382	0.64171	0.75576	0.892547
*Abcb1a*	0.004697	0.00501	0.006241	0.006113
*Abcc2*	0.280486	0.27168	0.2375	0.288371
*Aldh1a1*	0.056956	0.12587	0.133601	0.177513
*Angptl4*	0.102664	0.31208	0.393927	0.350139
*Anxa5*	0.300617	0.54715	0.527046	0.553249
*Atf3*	0.003511	0.02816	0.020992	0.020992
*Bhmt*	0.004322	0.00110	0.002349	0.002131
*Bmp1*	0.018149	0.05292	0.044378	0.035305
*Bmp4*	0.110798	0.09807	0.107024	0.128158
*Btg2*	0.022344	0.11744	0.104821	0.172659
*Calb1*	0.298541	0.07856	0.064974	0.088144
*Cat*	1.967004	0.85857	0.983502	0.936921
*Ccl3*	0.002606	0.00274	0.002176	0.001539
*Ccnd1*	0.357496	0.46329	0.407819	0.478304
*Ccng1*	0.462011	0.25000	0.44013	0.416388
*Ccs*	0.270931	0.08304	0.089374	0.10125
*Cd24a*	0.4222	1.15669	0.862144	0.862144
*Cd44*	0.008525	0.05995	0.046263	0.043767
*Cdkn1a*	0.013946	0.26981	0.428094	0.413512
*Clu*	0.270931	0.00083	6.390815	4.710891
*Cp*	0.038902	0.28917	0.205328	0.220065
*Cst3*	0.898755	0.70711	0.782412	0.844401
*Ctss*	0.045626	0.06515	0.110798	0.089374
*Cxcl10*	0.012396	0.02194	0.013754	0.015051
*Cxcl3*	0.00078	0.00155	0.001103	0.000848
*Cyp2c54*	0.001961	0.00164	0.001174	0.000981
*Cyp2d22*	0.023617	0.04039	0.034578	0.030522
*Cyr61*	0.047564	0.14459	0.110798	0.151354
*Egf*	1.145518	0.01269	0.011486	0.014438
*Fgb*	0.002754	0.48971	0.77164	0.495171
*Fmo2*	0.416388	0.10224	0.097801	0.133601
*Fn1*	0.019998	0.04389	0.0334	0.034339
*G6pc*	0.793334	0.01820	0.040554	0.047564
*G6pdx*	0.058965	0.10013	0.11793	0.126394
*Gadd45a*	0.103378	0.15283	0.171467	0.164482
*Gamt*	0.038634	0.00568	0.003462	0.003711
*Gatm*	0.990343	0.02896	0.036046	0.030734
*Gc*	0.022189	0.02683	0.032487	0.033169
*Ghr*	1.575708	0.50000	0.648869	0.592957
*Glul*	1.439931	0.82359	0.874179	0.75576
*Gpnmb*	0.002552	0.00626	0.005396	0.005743
*Gpx8*	0.052048	0.08479	0.074635	0.070609
*Gstk1*	0.19291	0.04039	0.050977	0.053142
*Gstp1*	0.592957	1.21419	0.997231	1.169588
*Havcr1*	0.132678	5.06303	5.759725	5.411393
*Hmox1*	0.052411	0.17924	0.166778	0.181243
*Hmox2*	0.12905	0.10882	0.113913	0.129947
*Hsp90aa1*	1.244874	0.80664	0.990343	0.990343
*Idh1*	1.121943	0.33448	0.710054	0.626767
*Igfbp1*	0.003121	0.00189	0.004965	0.005509
*Igfbp3*	0.804408	0.76313	0.495171	0.475
*Ipmk*	0.070121	0.06887	0.059788	0.073608
*Klk1*	4.910943	2.11404	1.597704	1.939924
*Lcn2*	0.022813	2.75108	2.743467	2.801113
*Lgals3*	0.17507	1.75321	1.352848	1.279872
*Mcm6*	0.048563	0.06887	0.08633	0.10125
*Mgp*	0.091887	0.14968	0.136408	0.112344
*Mt1*	0.326691	8.57419	9.421782	6.435267
*Nox4*	0.465225	0.08597	0.159984	0.157782
*Nphs2*	0.048901	0.05995	0.03972	0.047564
*Nqo1*	0.079992	0.28717	0.338212	0.380508
*Oat*	0.205328	0.14559	0.183773	0.162217
*Odc1*	3.773753	0.40613	0.597082	0.597082
*Rgn*	0.008765	0.01209	0.013946	0.01647
*Rtn4*	0.251042	0.65520	0.671752	0.690637
*Scd1*	0.179991	0.08778	0.181243	0.150309
*Slc22a1*	0.475	0.15073	0.130851	0.17507
*Slc22a5*	0.302708	0.13490	0.119576	0.132678
*Slc22a6*	1.420107	0.26243	0.309069	0.38582
*Socs3*	0.003276	0.06887	0.047235	0.050625
*Sod2*	1.07624	0.48633	0.446273	0.478304
*Sod3*	0.850274	0.82932	0.75054	1.004168
*Spp1*	2.355446	59.71411	84.80045	54.79605
*Sprr1a*	0.088144	0.36349	0.187635	0.171467
*Timp1*	0.003186	0.29937	0.2375	0.163346
*Tmsb10*	0.263523	1.56917	0.963262	0.911301
*Tnfrsf12a*	0.044687	0.75786	0.735094	0.924023
*Uchl1*	0.001518	0.00221	0.002146	0.002207
*Ugt1a1*	0.003056	0.00319	0.005069	0.005509
*Ugt1a6a*	0.049241	0.03955	0.024281	0.033633
*Vcam1*	0.006551	0.01618	0.01602	0.013659
*Vim*	0.052775	0.19345	0.185052	0.17507
*Actb*	2.899894	5.02805	5.084127	5.155099
*B2m*	1.011152	0.90752	1.106497	0.880259
*Gapdh*	3.044067	2.36199	3.06524	3.308093
*Gapdh*	0.080548	0.06381	0.050625	0.054259

Data presented as 2^ΔC_T_.

## Appendix F

**Table A4:** RT-PCR data from diet-FAN study.

	Chow		Western		Americanized		*p*	*p*	*p*
	Vehicle	FAN	Vehicle	FAN	Vehicle	FAN	(FAN)	(Diet)	(FAN X Diet)
**Kidney**									
Nephrotoxicity									
*Btg2*	1.0 ± 0.3	8.6 ± 2.0 ^ab^	1.2 ± 0.2	13.7 ± 4.4 ^a^	1.0 ± 0.26	7.0 ± 3.2 ^b^	<0.001	0.03	0.04
*Cdkn1a*	1.0 ± 0.1	20.7 ± 4.6 ^c^	0.5 ± 0.05	18.8 ± 8.7 ^b^	1.1 ± 0.17	44.0 ± 18.8 ^a^	<0.001	0.01	0.02
*Spp1*	1.0 ± 0.3	29.8 ± 8.2 ^b^	1.5 ± 0.5	88.6 ± 24.0 ^a^	2.7 ± 1.9	63.9 ± 17.7 ^b^	<0.001	0.001	0.001
*Tnfrsf12a*	1.0 ± 0.2	18.1 ± 6.9 ^a^	0.7 ± 0.1	11.4 ± 2.48.0 ^ab^	0.6 ± 0.12	9.4 ± 4.4 ^b^	<0.001	0.07	0.1
*Clu*	1.0 ± 0.1	42 ± 8.4	1.3 ± 0.2	55.2 ± 23.3	1.7 ± 0.58	73.4 ± 41.8	<0.001	NS	NS
*Fgb*	1.0 ± 0.2	53.7 ± 29.1	0.6 ± 0.2	70.9 ± 19.3	1.5 ± 2.06	191.3 ± 225.6	<0.001	NS	NS
Leukocyte Markers									
*Ccr2* (Monocytes)	1.0 ± 0.1	0.6 ± 0.1	1.5 ± 0.4	1.3 ± 0.7	1.5 ± 0.3	1.3 ± 1.1	NS	0.1	NS
*Cd3e* (T-cells)	1.0 ± 0.1	0.1 ± 0.1	0.9 ± 0.4	0.3 ± 0.2	1.2 ± 1.0	0.2 ± 0.3	<0.001	NS	NS
*Cd19* (B-cells)	1.0 ± 0.5	0.9 ± 0.3	1.9 ± 0.7	2.6 ± 1.1	1.7 ± 1.5	2.4 ± 1.4	<0.001	NS	NS
*Ly6g* (Neutrophils)	1.0 ± 1.9	36.6 ± 26.8	6.2 ± 6.1	34.8 ± 29.8	1.7 ± 3.4	61.3 ± 67.2	0.002	NS	NS
**Spleen**									
*Ccr2*	1.0 ± 0.2	1.1 ± 0.3	0.7 ± 0.3	0.7 ± 0.3	0.07 ± 0.3	0.8 ± 0.5	NS	NS	NS
*Cd3e*	1.0 ± 0.1	0.6 ± 0.2 ^a^	0.9 ± 0.3	0.4 ± 0.2 ^ab^	0.5 ± 0.05	0.4 ± 0.2 ^b^	0.001	0.01	NS
*Cd19*	1.0 ± 0.1	0.2 ± 0.07 ^a^	0.8 ± 0.3	0.02 ± 0.06 ^ab^	0.5 ± 0.07	0.1 ± 0.07 ^b^	<0.001	0.009	0.09
*Ly6g*	1.0 ± 0.1	2.9 ± 1.1	1.7 ± 0.3	5.9 ± 5.3	3.2 ± 2.5	2.0 ± 1.3	NS	NS	NS

Data are presented normalized to average of the Vehicle treated chow-fed group for each gene. Different superscript letters (a vs. b) signify significant difference between diets (*p* < 0.05) as determined by Two-Way ANOVA with Tukey Post Hoc test. Treatments with similar superscript letters are not statistically different (*p* > 0.05). Data analyzed with 2-Way ANOVA and Tukey post hoc analysis. ND-not detected, NS-*p* > 0.1.

## Appendix G

**Table A5:** RT-PCR data from diet-uIRI study.

	Chow		Western		Americanized		*p*	*p*	*p*
	Control	uIRI	Control	uIRI	Control	uIRI	(Ischemia)	(Diet)	(Ischemia X Diet)
**Kidney**									
Nephrotoxicity									
*Btg2*	1.0 ± 0.2	3.7 ± 1.9	0.6 ± 0.2	2.5 ± 0.5	0.9 ± 0.3	2.7 ± 0.8	<0.001	NS	NS
*Cdkn1a*	1.0 ± 0.6	4.3 ± 2.9	0.6 ± 0.5	5.3 ± 2.1	0.7 ± 0.3	6.0 ± 1.0	<0.001	NS	NS
*Spp1*	1.0 ± 0.4	12.9 ± 4.5	0.4 ± 0.3	11.6 ± 3.6	0.5 ± 0.2	15.2 ± 3.8	<0.001	NS	0.06
*Tnfrsf12a*	1.0 ± 0.4	2.9 ± 1.7	0.6 ± 0.2	2.7 ± 0.4	0.8 ± 0.3	3.7 ± 1.1	<0.001	NS	NS
*Clu*	1.0 ± 0.3	8.4 ± 5	0.7 ± 0.2	8.4 ± 2	0.8 ± 0.2	7.7 ± 2	<0.001	NS	NS
*Fgb*	1.0 ± 1.2	10.9 ± 10.6	0.5 ± 0.4	7.5 ± 6.6	0.6 ± 0.6	13.1 ± 5	<0.001	NS	NS
Leukocyte Markers									
*Ccr2* (Monocytes)	1.0 ± 0.3	7.3 ± 6.2	0.9 ± 0.2	4.5 ± 1.4	1.2 ± 0.5	4.4 ± 1.8	<0.001	NS	NS
*Cd3e* (T-cells)	1.0 ± 0.6	1.4 ± 0.5	1.1 ± 0.5	1.9 ± 0.9	1.6 ± 1.1	2.0 ± 0.2	0.1	NS	NS
*Cd19* (B-cells)	1.0 ± 0.9	2.6 ± 1.9 ^b^	1.0 ± 0.5	8.6 ± 4.5 ^ab^	1.5 ± 1.4	8.4 ± 1.4 ^a^	<0.001	0.04	0.02
*Ly6g* (Neutrophils)	1.0 ± 1.0	18.8 ± 14.5	0.5 ± 0.4	10.6 ± 5.7	0.3 ± 0.3	5.5 ± 3.2	<0.001	0.06	NS
**Spleen**									
*Ccr2*	-	1.0 ± 0.4 ^a^	-	0.6 ± 0.2 ^b^	-	0.6 ± 0.3 ^b^	-	<0.004	-
*Cd3e*	-	1.0 ± 0.2	-	1.1 ± 0.2	-	0.9 ± 0.3	-	0.06	-
*Cd19*	-	1.0 ± 0.2 ^a^	-	0.9 ± 0.2 ^ab^	-	0.6 ± 0.09 ^b^	-	0.008	-
*Ly6g*	-	1.0 ± 0.9 ^a^	-	0.6 ± 0.2 ^ab^	-	0.2 ± 0.1 ^b^	-	0.03	-

Data are presented normalized to average of data from the non-ischemic contralateral kidney of the chow-fed group for each gene. Different superscript letters (a vs. b) signify significant difference between diets (*p* < 0.05) as determined by Two-Way ANOVA with Tukey Post Hoc test. Treatments with similar superscript letters are not statistically different (*p* > 0.05). Data analyzed with 2-Way ANOVA and Tukey post hoc analysis. NS, *p* > 0.1.
